# The economic and environmental effects of China’s environmental expenditure under financing constraints

**DOI:** 10.1371/journal.pone.0305246

**Published:** 2024-07-12

**Authors:** Ruiliang Wang, Jie Yan, Wenfu Wang

**Affiliations:** 1 School of Public Finance and Taxation, Southwestern University of Finance and Economics, Chengdu, China; 2 School of Economics, Sichuan University, Chengdu, China; Zhanjiang University of Science and Technology, CHINA

## Abstract

Sound ecological and environmental governance systems are critical for promoting green and low-carbon economic transformation and high-quality development. However, financing constraints are major obstacle to the revitalization and transformation of China’s real economy. In this study, we constructed an environmental dynamic stochastic general equilibrium (E-DSGE) model that incorporates two types of environmental expenditure and financing constraints, and discussed their economic and environmental effects. Based on this, we further considered the impacts of financing constraints on policy effects. Firstly, we found that increases in carbon emission reduction subsidies in government expenditure (1) increase total economic output and (2) motivate enterprises to increase emission reduction efforts and reduce pollution intensity and emissions, thereby reducing the inventory of environmental pollutants while balancing economic benefits and emission reduction. Secondly, increasing the proportion of government special expenditure on environmental protection promote output growth and directly reduces the pollution stock in the environment. However, such policies may also reduce the emission reduction efforts of enterprises, leading to increases in their pollution emissions and intensity. Lastly, the existence of financing constraints is not conducive to the growth of total output but increases the pollution control effect of emission reduction subsidies and pollution prevention expenditure. Application of the E-DSGE model offers new theoretical insight into environmental economics and macroeconomics. Moreover, the results of this study provide a reference for optimizing the structure of fiscal expenditure.

## 1. Introduction

As the largest developing country, China faces concurrent challenges of developing the economy, improving people’s livelihoods, eradicating poverty, and controlling pollution. In addition, reaching a new carbon peak and achieving carbon neutrality are significant challenges. In recent years, with the strengthening of China’s environmental governance, significant progress has been made in reducing pollution and carbon emissions. However, resource and environmental constraints accumulated during long-term rapid industrialization are becoming increasingly prominent. On March 11, 2021, the fourth session of the 13^th^ National People’s Congress (NPC) passed a resolution on the 14^th^ Five-Year Plan for National Economic and Social Development and the Outline for the Long-Range Objective for 2035, which put forward the general requirements of “thoroughly fighting the battle of pollution prevention and control, establishing a sound environmental governance system, promoting precise, scientific, legal, and systematic pollution control, collaborating to reduce pollution and carbon emissions, continuously improving air and water quality, and effectively controlling the risk of soil pollution.” To achieve this, China must establish sound ecological and environmental governance systems, innovate environmental governance tools, improve environmental quality, and promote green and low-carbon economic transformation and high-quality development. Environmental expenditure is an important measure for assisting ecological environment governance and promoting the green and low-carbon transformation of the economy and society. On one hand, by controlling environmental pollution, the concentration of pollution in the environment can be reduced, and the ecological environment can be improved; on the other hand, by providing emission reduction subsidies to enterprises, they can be guided to engage in green and low-carbon production, promoting green and low-carbon transformation of the economy and society. China’s environmental expenditure has been continuously increasing in recent years; however, the underlying mechanisms of its macroeconomic and environmental effects require further research. Moreover, financing constraints have long been a major obstacle to the revitalization and transformation of China’s real economy.

Research by the World Bank has shown that financing constraints are a key factor in the development of non-financial listed enterprises in China, especially private and medium-sized enterprises that face long-term financing difficulties [1, 2]. Moreover, after the COVID-19 pandemic, logistics interruptions seriously affected the production and sales channels of enterprises, causing serious economic losses [3]. With declining revenue and losses, financing constraints have become increasingly serious. Previous studies have shown that the epidemic affected the loan decisions of banks and other financial institutions through channels such as risk aversion and financing liquidity, reducing their credit supply to enterprises [4–9]. Although fintech companies expanded their loans to financially constrained enterprises during this period, the increase in credit supply was unsustainable, and financing constraints remain serious [10]. This is because methods such as information reminders, triggering social pressure, and economic incentives have reduced the default rate of loans, but default events still occur frequently, making this type of loan unsustainable [11]. Recent research also found that financing constraints have varying degrees of impact on the effects of various environmental policies, resulting in unsatisfactory policy outcomes [12]. This is because the implementation of environmental and financial policies belongs to different administrative departments, resulting in insufficient synergy between finance and environmental policies [13]. However, how financing constraints impact on emissions and the pollution reduction effects of environmental expenditure remains unclear.

It is difficult to solve environmental pollution problems solely by relying on the market itself; the participation and leadership of the government is also needed. Fiscal expenditure is an important economic tool for government demand-side management and an important factor influencing environmental quality [14, 15]. Environmental expenditure refers to the financial expenditure carried out by the government to provide green environmental protection products and services and meet the common environmental needs of society. This is the most direct and effective way for governments to participate in environmental pollution control.

The impact of environmental expenditure on environmental pollution is reflected in both direct and indirect effects. Direct effects, which include input-based environmental regulation, play a role in environmental governance, reducing pollution emissions and environmental pollutants, and improving environmental quality [16]. Erdmenger [17] found that government spending on green products and services is the most direct and effective way to address environmental problems and pollution. Bostan et al. [18] found that government environmental investment can significantly reduce the emissions of harmful gases from polluting sectors.

Indirect effects, which include increasing the proportion of investment in clean elements and introducing and developing advanced emission reduction technologies, can provide a good external environment for enterprises to actively reduce emissions and transform production. Jiang [16] found that environmental expenditure can promote regional pollution reduction by inducing social capital and stimulating technological innovation. Delong and Summers [19] and Antoci et al. [20] suggest that the best approach for government environmental expenditure policies is to subsidize and support high-tech industries directly related to environmental technology research & development (R&D), reduce enterprise R&D costs, and obtain positive externalities in technology R&D. Li and Huang [21] found that environmental expenditure not only has a significant inhibitory effect on local carbon emissions but also a spatial spillover effect, reducing carbon emissions in surrounding areas. Other studies have examined the positive incentive effect of environmental protection expenditure and emission reduction subsidies on pollution reduction by polluting enterprises [22–28]. Chu et al. [22] and Yi and Li [24] found that providing emission reduction subsidies to the coal industry can reduce carbon emissions. Ai et al. [25] showed that desulfurization electricity price subsidy policies for coal-fired power plants can significantly reduce emissions. Studies based on dynamic stochastic general equilibrium models by Wu [26] and Cai et al. [27] also support this view.

Previous studies have also shown that environmental expenditure has significantly impacts structural economic transformation and growth [16, 29–33]. On one hand, environmental expenditure can induce social capital to invest in the environment, reducing environmental pollution and causing changes in the economic structure. Ruffing [34] found that environmental spending reduces air pollution and stimulates environmental financing and investment. Tian et al. [35] found that environmental expenditure guides the direction, scale, and structure of nongovernmental investment through subsidies and depreciation policies, thereby causing changes in the economic structure. Benson et al. [36] suggest that environmental governance investment is only effective in achieving green growth through effective cooperation with enterprises by acting on their green technology R&D investment activities. Sun and Zhao [30] revealed a long-term dynamic relationship between environmental protection investment and economic transformation, and that the two promote each other. On the other hand, environmental expenditure can have a positive impact on economic growth on both the demand and supply sides. On the demand side, Jiang [16] constructed an input–output model for the contribution of environmental protection investment to the economy and found that environmental protection investment expanded domestic demand and had a significant driving effect on economic growth. Li and Cheng [37] found that government capital environmental protection expenditure not only plays a front-end role in pollution prevention and control of environmental governance, but also promotes enterprise value enhancement by stimulating innovation and reputation when the expenditure level reaches a certain scale, achieving a win–win situation of environmental protection and economic benefits. On the supply side, Jiang [16] found that environmental subsidies can promote regional economic development by inducing social capital and stimulating technological innovation. Tang and Yang [38] found that both R&D and non-R&D financial environmental subsidies can promote green technology innovation in enterprises, contributing to the improvement of sustainable development performance. Jiang et al. [39] found that environmental expenditure can reduce agricultural non-point source pollution and improve agricultural technology progress, thereby leading to an improvement in agricultural ecological efficiency.

The Dynamic Stochastic General Equilibrium (DSGE) model can provide a strong and consistent framework for policy discussion and analysis, help identify the sources of economic fluctuations, evaluate the potential effects of different policies, and have predictive power comparable to traditional macroeconomic models. We introduce environmental variables into the DSGE model to form the Environmental Dynamic Stochastic General Equilibrium (E-DSGE) model. The existing literature is focused on the environmental effects of environmental expenditure; however, there is relatively little research on macroeconomic effects and their mechanisms of action. Moreover, existing research mostly focuses on a certain type of environmental expenditure or total environmental expenditure, but does not include different types of environmental expenditure in a unified analytical framework. Finally, although scholars [26–28] have used the Dynamic Stochastic General Equilibrium (DSGE) model to study the impact of environmental expenditure on environmental quality, there has been no further exploration of financing constraints. Moreover, these studies often use corporate emission reduction efforts as exogenous variables, making it difficult to reflect the internal mechanism of environmental expenditure on pollution reduction.

To address these gaps in knowledge, in this study, we examined the macroeconomic effects of environmental expenditure, explored the impact and mechanism of environmental expenditure on emissions and pollution reduction, and analyzed the impact of financing constraints on policy effectiveness. This study makes three main contributions. (1) Based on the background of financing constraints commonly faced by Chinese enterprises, this study analyzed the environmental effects of environmental expenditure and its macroeconomic effects, enriching theoretical research in the field of green finance. (2) This study provides in-depth analysis on the policy effects of two types of environmental expenditure (emission reduction subsidies and pollution prevention and control expenditure), which can provide a reference for governments to optimize fiscal expenditure structure. (3) Finally, this study developed an Environmental Dynamic Stochastic General Equilibrium (E-DSGE) model with financing constraints and two types of environmental expenditure. E-DSGE can analyze the mechanisms by which these types of expenditure affect macroeconomics and environmental pollution, enriching the theoretical research on macroeconomics.

## 2. Experiential evidence

As mentioned above, financing constraints may affect environmental spending policies to varying degrees [12, 13]. In this section, we will take environmental subsidies as an example to empirically test the relationship between environmental expenditure, financing constraints and enterprise output, so as to provide a practical basis for the dynamic simulation and mechanism of action test using DSGE model below.

### 2.1. Data sources

We select the data of Chinese traditional manufacturing enterprises from 2016 to 2021 to discuss the relationship between financing constraints, environmental protection subsidies and enterprise output, and test the regulatory role of financing constraints. The data presented in this paper are mainly obtained from the WIND database in China.

### 2.2. Construction of the econometric model

To test the relationship between financing constraints, environmental subsidies and enterprise output, we set the following regression model, as shown in Eqs (1)–(4).


$$Output_{i,t}=m_{0}+m_{1}KZ_{i,t}+m_{k}\sum Control+\sum Year+\sum Firm+\sum Industry+\varepsilon_{i,t}$$ (1)

$$Output_{i,t}=n_{0}+n_{1}Greensubsidy_{i,t}+n_{k}\sum Control+\sum Year+\sum Firm+\sum Industry+\varepsilon_{i,t}$$ (2)

$$Output_{i,t}=l_{0}+l_{1}KZ_{i,t}+l_{2}Greensubsidy_{i,t}+l_{k}\sum Control+\sum Year+\sum Firm+\sum Industry+\varepsilon_{i,t}$$ (3)

$$\begin{array}{l} Output_{i,t}=\gamma_{0}+\gamma_{1}KZ_{i,t}+\gamma_{2}Greensubsidy_{i,t}+\gamma_{3}KZ_{i,t}\times Greensubsidy_{i,t}+\gamma_{k}\sum Control+\sum Year+\\ \;\sum Firm+\sum Industry+\varepsilon_{i,t} \end{array}$$ (4)

Among them, *Output* represents the output of the enterprise, with the enterprise operating income; *KZ* represents the financing constraint, the greater the value, the greater the financing constraint [40]. *Greensubsidy* represents the environmental subsidy, with the log value of the amount of the environmental subsidy. ∑*Control*_*it*_ represents the set of control variables, including enterprise size (*Size*) enterprise age (*Age*); research and development expenditure (*RD*); corporate liabilities (*Debtrate*); proportion of current assets (*Current*); equity concentration (*Ownership*); cash dividend (*Cashdividends*); institutional shareholding (*IS*), digital transformation (*DT*). Among them, the enterprise digital transformation variable is expressed by the digital word frequency data [41]. Moreover, ∑*Year* represents the time fixed effect, ∑*Firm* represents the individual fixed effect, ∑*Industry* is the industry fixed effect, and *ε*_*it*_ represents the random disturbance term.

### 2.3. Empirical analysis results

The relationship between environmental protection subsidies, financing constraints and enterprise output is shown in Table 1. In Table 1, columns (1) and (2) show the estimated results of the impact of financing constraints on enterprise output. It can be seen that financing constraints have a negative impact on enterprise output, whether control variables are added. We found that the environmental subsidy variable (*Greensubsidy*) was endogenous, which interfered with the estimation results of the model. The specific approach is to use the lag phase of *Greensubsidy* as the instrumental variable and use the two-stage least squares method for estimation. Therefore, we used the lag phase of *Greensubsidy* as the instrumental variable and used the two-stage least squares method (IV-2 SLS) for estimate.

**Table 1:** Environmental protection subsidies, financing constraints and enterprise output.

Variables	(1)	(2)	(3)	(4)	(5)	(6)	(7)
KZ	-0.00670**	-0.00596***		-0.0205***	-0.0147***	-0.0130**	-0.0147***
	(0.00291)	(0.00210)		(0.00518)	(0.00560)	(0.00547)	(0.00559)
Greensubsidy			0.00847***	0.00841***	0.0105***	0.0106***	0.0106***
			(0.00191)	(0.00190)	(0.00238)	(0.00253)	(0.00257)
Greensubsidy×KZ					-0.00197***	-0.00198***	-0.00156**
					(0.000700)	(0.000751)	(0.000752)
Control Variables	NO	YES	YES	YES	YES	YES	YES
Constant	16.55***	-2.716***	-6.364***	-6.372***	-6.392***	-6.530***	-6.495***
	(0.0359)	(0.204)	(0.162)	(0.171)	(0.170)	(0.176)	(0.175)
Time	YES	YES	YES	YES	YES	YES	YES
Individual	YES	YES	YES	YES	YES	YES	YES
Industry	YES	YES	YES	YES	YES	YES	YES
Observations	8,173	8,136	6,791	6,791	6,791	5,861	5,552
R-squared	0.306	0.697	0.887	0.888	0.887	0.881	0.884

Standard errors in parentheses

*** p<0.01

** p<0.05

* p<0.1

Column (3) shows the impact of environmental subsidies on enterprise output. It can be seen that the coefficient of variable *Greensubsidy* is positive, indicating that environmental subsidies have a promoting effect on enterprise output. Column (4) shows the estimation results of adding financing constraint variable and environmental subsidy variable at the same time. The results show that financing constraint has a negative impact on enterprise output, while environmental subsidy has a positive impact. Column (5) shows the estimated results after continuing to add the cross term of financing constraints and environmental subsidies, and finds that financing constraints have a negative adjustment effect on the policy effect of environmental subsidies. Column (6) and column (7) are the estimation results after excluding the samples of provincial cities and first-tier cities, respectively. The symbols of each major variable remain unchanged, indicating a good robustness of the model.

## 3. Model construction

Empirical research in Section 2 suggests that environmental spending may have a positive impact on economic output, while financing constraints may lead to unsatisfactory policy effects. Next, we will build a DSGE model for dynamic simulation and the mechanism of action test.

**Fig 1:**
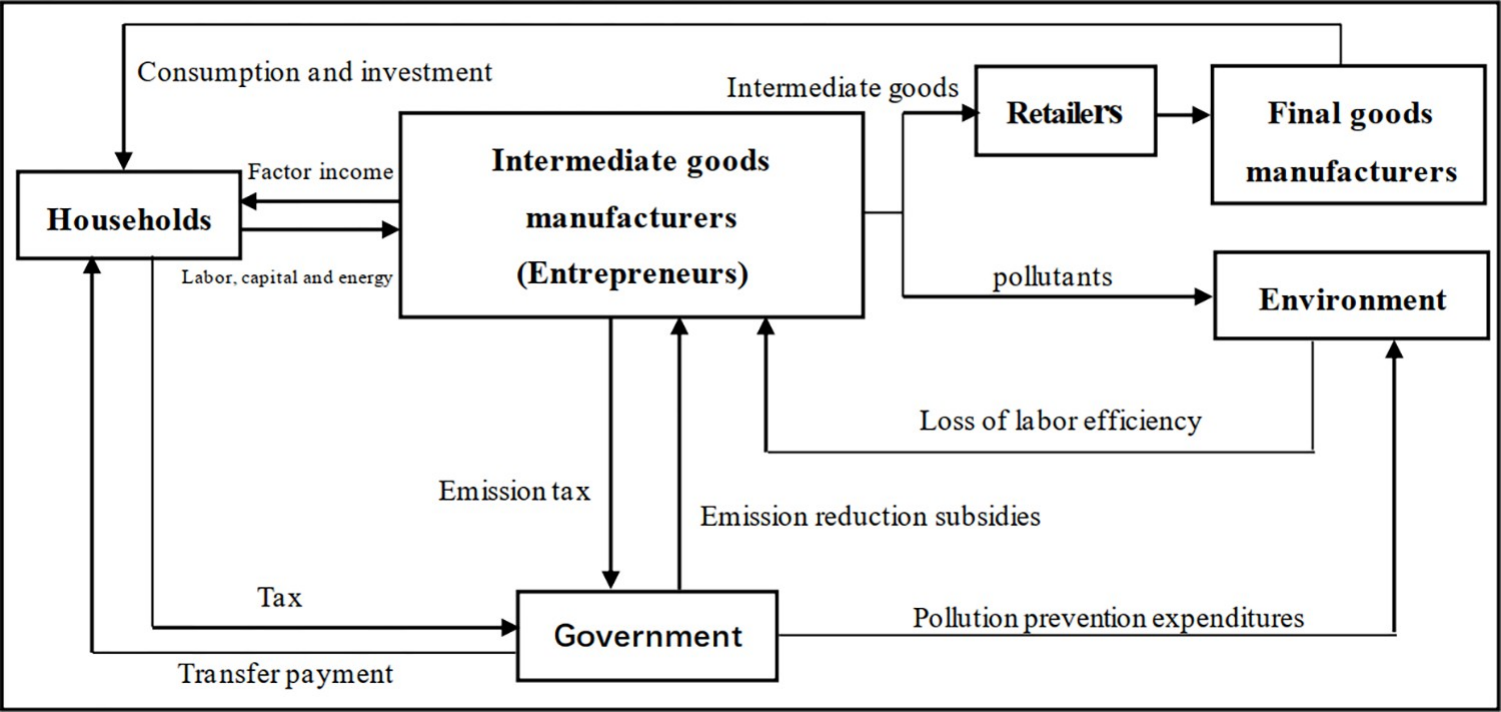
Basic framework of the E-DSGE model.

We constructed an E-DSGE model that includes two types of environmental expenditure and financing constraints. This economic system includes six sectors: households, financial intermediaries, intermediate manufacturers, retailers, final goods manufacturers, and the government. The structure of the model is as follows. Households are assumed to be the owners of the factors of production, and they receive factor income by providing labor, net investment, and energy to intermediate manufacturers; interest income from deposits with financial intermediaries; and transfers from the government, which they then use for consumption, investment, and taxation. Financial intermediaries obtain deposits from residents and use these to provide loans to governments and entrepreneurs. Intermediate manufacturers in a perfectly competitive relationship use three factors—capital, labor, and energy—to produce intermediate goods, and emit carbon dioxide into the environment. Retailers with monopolistic competition purchase intermediate goods from intermediate manufacturers, repackage them, and label them for sale to final goods manufacturers. The final goods manufacturers in a fully competitive relationship purchase packaged intermediate goods from retailers and use assembly techniques to produce the final goods. Finally, the government generates revenue through labor and emission taxes and borrowing from financial intermediaries, which is used for carbon emission reduction subsidies to intermediate manufacturers, special expenditure on environmental protection, transfer payments to residents, and debt interest repayment. Assuming that resident deposits flow exclusively to the government and entrepreneurs, it can be understood that households provide loans directly to the government and enterprises, causing the omission of the analysis of the financial sectora. A simplified model framework is illustrated in Fig 1.

### 3.1. Households

Suppose there are a large number of homogeneous and infinitely living households in the economy; representative households derive positive utility from consumption and negative utility from labor and carbon emissions, and the household’s decision is to maximize the discounted value of expected real utility with an objective function of:

$$\underset{\left\{C_{t}^{h},L_{t},CO_{2t},D_{t},I_{1}\right\}}{\max}E_{t}\sum_{t=0}^{\infty }{\left(\beta_{h}\right)}^{t}\{\ln C_{t}^{h}-\mu_{L}\frac{{L_{t}}^{1+\eta }}{1+\eta }-\mu_{E}\frac{C{O_{2t}}^{1+\nu }}{1+\nu }\}$$ (5)

where *E*_*t*_ refers to an operational symbol; *β*_*h*_ is the subjective discount factor of households; $C_{t}^{h}$ is the actual consumption of households, which is the value of nominal consumption after excluding price inflation factors; *L*_*t*_ is labor supply, which refers to the labor factors provided by households to the production sector; *CO*_2*t*_ is the carbon emissions of enterprises, which is a function of the degree of emission reduction effort *e*_*t*_ and energy input *EN*_*t*_ of the intermediate manufacturer (i.e., $CO_{2t}=(1-e_{t})\varphi EN_{t}$); *η* is the elasticity of household labor supply; *ν* refers to the elasticity of the energy supply; and *μ*_*L*_ and *μ*_*E*_ are negative utility coefficients for labor and pollution emissions, respectively.

The budget constraint for households is:

$$C_{t}^{h}+D_{t}+I_{t}=\frac{R_{t-1}^{d}D_{t-1}}{\pi_{t}}+(1-\tau^{L})w_{t}L_{t}+(1-\tau^{K})q_{t}^{K}I_{t}^{N}+(1-\tau^{E})p_{t}^{E}EN_{t}+\sum_{t}+G_{t}^{T}$$ (6)

where *D*_*t*_ is bank deposits held by a household, and $R_{t}^{d}$ is the deposit interest rate; *w*_*t*_ is the actual labor wage rate; $I_{t}^{N}$ is the net investment for a household, and $q_{t}^{K}$ is the actual price of the net investment; $p_{t}^{E}$ is the price of energy; *π*_*t*_ is the inflation rate; ∑_*t*_ is the profit from the enterprise; $G_{t}^{T}$ represents the government’s transfer payment to the household sector, which are government support expenditures for specific groups such as the poor and vulnerable; and *τ*^*L*^, *τ*^*K*^, and *τ*^*E*^ represent the labor, capital, and energy tax rates, respectively.

In addition, the equation for net investment is as follows:

$$I_{t}^{N}=I_{t}-\frac{\psi_{I}}{2}{\left(\frac{I_{t}}{I_{t-1}}-1\right)}^{2}I_{t}$$ (7)

where $\frac{\psi_{I}}{2}{\left(\frac{I_{t}}{I_{t-1}}-1\right)}^{2}I_{t}$ represents the investment adjustment cost and *ψ*_*I*_ is the investment adjustment cost factor.

By constructing and solving the Lagrangian function, the following first-order optimality conditions are obtained:

$${\left(C_{t}^{h}\right)}^{-1}=\lambda_{t}^{h}$$ (8)

$$\mu_{L}{\left(L_{t}\right)}^{\eta }=\lambda_{t}^{h}(1-\tau^{L})w_{t}$$ (9)

$$\mu_{E}{\left[(1-e_{t})\varphi EN_{t}\right]}^{1+\nu }=\lambda_{t}^{h}(1-\tau^{E})EN_{t}{p_{t}}^{E}$$ (10)

$$1=\beta_{h}E_{t}\frac{\lambda_{t+1}^{h}}{\lambda_{t}^{h}}\frac{R_{t}^{d}}{\pi_{t+1}}$$ (11)

$$\frac{1}{q_{t}^{K}(1-\tau^{K})}=1-\psi_{I}(\frac{I_{t}}{I_{t-1}}-1)(\frac{I_{t}}{I_{t-1}})-\frac{\psi_{I}}{2}{\left(\frac{I_{t}}{I_{t-1}}-1\right)}^{2}+\beta_{h}E_{t}\frac{\lambda_{t+1}^{h}}{\lambda_{t}^{h}}\frac{q_{t+1}^{K}}{q_{t}^{K}}\psi_{I}(\frac{I_{t+1}}{I_{t}}-1){\left(\frac{I_{t+1}}{I_{t}}\right)}^{2}$$ (12)

### 3.2. Enterprises

The model constructed in this study includes three types of enterprise: intermediate manufacturers, retailers, and final goods manufacturers. The retail market is monopolistically competitive with price stickiness, whereas the intermediate goods and final goods markets are perfectly competitive.

#### 3.2.1. Final goods manufacturers

The final goods manufacturer buys packaged intermediate goods from the retailer and produces final goods through a combination of technologies. The production function of the final goods manufacturer is a common constant elasticity of substitution (CES) function of the form: $Y_{t}=[\int_{0}^{1}{y_{j,t}}^{\frac{\theta -1}{\theta }}dj{]}^{\frac{\theta }{\theta -1}}$. Where *Y*_*t*_ is the final output, *y*_*j*,*t*_ is intermediate goods purchased from retailer *j*, and *θ* is the elasticity of substitution between intermediate goods. The profit maximization of final product manufacturers is as follows:

$$\underset{y_{j,t}}{\max}P_{t}{\left[\int_{0}^{1}{y_{j,t}}^{\frac{\theta -1}{\theta }}dj\right]}^{\frac{\theta }{\theta -1}}-\int_{0}^{1}P_{j,t}y_{j,t}dj$$ (13)

where *P*_*j*,*t*_ is the price of intermediate goods after retailer *j* and *P*_*t*_ is the general price level. The demand for intermediate goods *j* and general aggregate price level *P*_*t*_ are obtained by solving the above maximization problem:

$$y_{j,t}={\left(\frac{P_{j,t}}{P_{t}}\right)}^{-\theta }Y_{t}$$ (14)

$$P_{t}={\left[{\int_{0}^{1}P_{j,t}}^{1-\theta }dj\right]}^{\frac{1}{1-\theta }}$$ (15)

Drawing on Calvo’s pricing approach, assume that only a proportion of 1-*ξ* retailers can adjust their prices in each period of the economy, and the remaining proportion of *ξ* retailers can only set their prices according to the previous period’s prices. After derivation, the equation of motion for inflation is obtained as:

$$1=\xi {\pi_{t}}^{\theta -1}+(1-\xi ){\left(\pi_{t}^{*}\right)}^{1-\theta }$$ (16)

Where $\pi_{t}^{*}$ is the variable in the optimal pricing conditions for retailers.

#### 3.2.2. Retailers

The optimization of a retailer who buys intermediate goods from an intermediate manufacturer and sells them to a final goods manufacturer is given by:

$$\underset{P_{j,t}}{\max}E_{t}\sum_{s=0}^{\infty }{\left(\beta_{h}\xi \right)}^{i}\lambda_{t+s}^{h}(\frac{P_{j,t}-P_{t+s}mc_{j,t+s}}{P_{t+s}})y_{j,t+s}$$ (17)

where *mc*_*j*,*t*_ is the relative price of the intermediate goods. By substituting Formula (10) into Formula (13) and solving it, we can obtain the optimal pricing condition for the retailer under sticky price conditions.


$$\pi_{t}^{*}=\frac{\theta }{\theta -1}\frac{E_{t}\sum_{s=0}^{\infty }{\left(\beta_{h}\xi \right)}^{s}\lambda_{t+s}^{h}mc_{t+s}{\left(\frac{P_{t+s}}{P_{t}}\right)}^{\theta }Y_{t+s}}{E_{t}\sum_{s=0}^{\infty }{\left(\beta_{h}\xi \right)}^{s}\lambda_{t+s}^{h}{\left(\frac{P_{t+s}}{P_{t}}\right)}^{\theta -1}Y_{t+s}}$$ (18)

#### 3.2.3. Intermediate manufacturers

Assume that the intermediate goods market is perfectly competitive, and all intermediate manufacturers are homogeneous. Intermediate manufacturer *i* produces intermediate goods by investing in capital *K*_*i*,*t*−1_, labor *L*_*i*,*t*_, and energy *EN*_*i*,*t*_, and its production function adopts the form of a Cobb–Douglas (C–D) function. The negative externality of carbon emissions is reflected in its effect on labor efficiency. Drawing on Xiao et al. [42], the production function is set to the following form:

$$y_{i,t}=A_{t}{\left(K_{i,t-1}\right)}^{\alpha_{1}}{\left(e_{t}^{L}L_{i,t}\right)}^{\alpha_{2}}{\left(EN_{i,t}\right)}^{1-\alpha_{1}-\alpha_{2}}$$ (19)

where *α*_1_ and *α*_2_ are the output shares of physical capital and labor of intermediate manufacturers, respectively; *A*_*t*_ represents total factor productivity; $e_{t}^{L}L_{i,t}$ is effective labor; $e_{t}^{L}$ is labor efficiency, which is a quadratic function of environmental pollution stock (*CS*_*t*_). Referring to Heutel [43], Annicchiarico and Di Dio [44], the labor efficiency ($e_{t}^{L}$) function is set as follows:

$$e_{t}^{L}=1-\Gamma (CS_{t})$$ (20)

where Γ(*CS*_*t*_) indicates the degree of the negative externality of carbon emissions, which is a quadratic function of pollution stock, and $\Gamma (CS_{t})=d_{0}+d_{1}CS_{t}+d_{2}{\left(CS_{t}\right)}^{2}$.

Referring to Xu et al. [45], the pollution stock (*CS*_*t*_) is set as a function of carbon emissions and special government expenditure on environmental protection, and the equation of motion of the pollution stock can be simply expressed as follows:

$$CS_{t}=(1-\delta_{CS})CS_{t-1}+CO_{2t}-\chi G_{t}^{E}$$ (21)

where *CS*_*t*−1_ is the pollution stock of the previous period, *CO*_2*t*_ is current carbon emissions, $G_{t}^{E}$ is the government’s special expenditure on environmental protection, *δ*_*CS*_ is the natural attenuation coefficient of carbon pollutants, *χ* is the effectiveness coefficient of government spending on environmental protection, and 0≤*δ*_*X*_≤1 and *χ*≥0.

In addition, capital (*K*_*i*,*t*_) is accumulated from net investment ($I_{i,t}^{N}$), and the capital movement equation is shown in Eq (19):

$$K_{i,t}=(1-\delta_{K})K_{i,t-1}+I_{i,t}^{N}$$ (22)

where *δ*_*K*_ is the capital depreciation rate.

Assuming that the carbon emissions of manufacturer *i* are *CO*_2*i*,*t*_,which is a certain percentage of its energy input *EN*_*i*,*t*_ and is negatively correlated with the enterprise’s effort to reduce emissions *e*_*i*,*t*_ [46], the carbon emissions of manufacturer *i* can be expressed as:

$$CO_{2i,t}=(1-e_{i,t})\varphi EN_{i,t}$$ (23)

where *φ* is the carbon emission factor per unit of energy input.

To analyze the environmental load of carbon emission reduction subsidies on unit output, an indicator of carbon emission intensity (*EI*_*t*_) is introduced, and the carbon emission intensity of intermediate manufacturers *i* is:

$$EI_{i,t}=CO_{2i,t}/y_{i,t}$$ (24)

According to the principle of “polluter pays,” the government levies a carbon tax on the emissions of intermediate manufacturers. Assuming that the effective rate of the carbon tax is $\tau_{t}^{CO_{2}}$, then the carbon tax ($tax_{t,i}^{CO_{2}}$) paid by the intermediate manufacturers *i* is:

$$tax_{t,i}^{CO_{2}}=\tau_{t}^{CO_{2}}CO_{2i,t}=\tau_{t}^{CO_{2}}(1-e_{i,t})\varphi EN_{i,t}$$ (25)

Simultaneously, to incentivize intermediate manufacturers to save energy and reduce carbon emissions, the government subsidizes carbon emission reduction practices. Assuming that the carbon emission reduction subsidies given by the government to intermediate manufacturers is $G_{i,t}^{sub}$, which is positively correlated with the rate of carbon emission reduction subsidies ($p_{t}^{sub}$) and the degree of carbon emission reduction efforts (*e*_*i*,*t*_), and negatively correlated with the carbon emissions (*φEN*_*i*,*t*_), the carbon emission reduction subsidies received by intermediate manufacturers can be expressed as:

$$G_{i,t}^{sub}=p_{t}^{sub}e_{i,t}{\left(\varphi EN_{i,t}\right)}^{-1}$$ (26)

The emission reduction efforts of intermediate manufacturers include the use of low-carbon technologies, installation and upgrading of carbon emission reduction equipment, and improvement of production management practices, all of which entail certain emission reduction costs. Drawing on Wang et al. [46], the carbon emission reduction costs of intermediate manufacturers were set as a function of the degree of emission reduction efforts and energy inputs, as follows:

$$ERC_{i,t}=\phi_{1}{\left(e_{i,t}\right)}^{\phi_{2}}EN_{i,t}$$ (27)

where *ϕ*_1_ and *ϕ*_2_ are technical parameters of enterprise emission reduction cost, and *ϕ*_1_>0, *ϕ*_2_>0.

Assuming that entrepreneurs own intermediate production enterprises and obtain utility through consumption. The utility function of entrepreneur *i* is given by:

$$E_{t}\sum_{t=0}^{\infty }{\left(\beta_{e}\right)}^{t}\ln C_{i,t}^{e}$$ (28)

Where $C_{i,t}^{e}$ is actual consumption of the entrepreneur *i*, and *β*_*e*_ represents the subjective discount factor for entrepreneurial consumption.

The financial constraints that the entrepreneur *i* must meet are:

$$mc_{i,t}y_{i,t}+b_{i,t}^{e}=C_{i,t}^{e}+b_{i,t-1}^{e}\frac{R_{t-1}^{d}}{\pi_{t}}+w_{t}L_{i,t}+q_{t}^{k}I_{i,t}^{N}+p_{t}^{E}EN_{i,t}+\phi_{1}{\left(e_{i,t}\right)}^{\phi_{2}}EN_{i,t}+tax_{t,i}^{CO_{2}}-p_{t}^{sub}e_{i,t}{\left(\varphi EN_{i,t}\right)}^{-1}$$ (29)

In Formula (29), the left side represents the income item and the right side represents the expenditure item. Where *mc*_*i*,*t*_ is the relative price between the market price and the final price and $b_{i,t}^{e}$ is the enterprise loan.

Financing constraints are a common phenomenon faced by Chinese enterprises in the development process and are one of the main factors hindering such development. Taking cues from Kiyotaki and Moore [47], we impose financing constraints on entrepreneurs’ financing activities. We assume that the entrepreneur obtains a loan by pledging his capital as collateral, and that if he fails to meet his commitment to repay the debt, the financial intermediary has the right to confiscate his assets as a percentage of the expected value of the capital; that is, $(1-m_{b})E_{t}q_{t+1}^{K}K_{i,t}$. The financing constraint faced by the entrepreneur can be expressed as:

$$b_{i,t}^{e}\leq m_{b}E_{t}\frac{q_{t+1}^{K}K_{i,t}\pi_{t+1}}{R_{t}^{d}}$$ (30)

where $b_{i,t}^{e}$ is the maximum loan received by the entrepreneur, *m*_*b*_≤1 is the degree of financing constraints faced by entrepreneurs in the borrowing process, and the smaller the value, the greater the financing constraint.

The first-order conditions for $C_{i,t}^{e}$, $b_{i,t}^{e}$, *L*_*i*,*t*_, *K*_*i*,*t*_, *EN*_*i*,*t*_, and *e*_*i*,*t*_ were obtained by solving the entrepreneur’s utility maximization problem:

$${\left(C_{i,t}^{e}\right)}^{-1}=\lambda_{t}^{e}$$ (31)

$$\lambda_{t}^{e}=\beta_{e}\lambda_{t+1}^{e}\frac{R_{t}^{d}}{\pi_{t+1}}+\lambda_{t}^{b}$$ (32)

$$w_{t}=\alpha_{2}mc_{i,t}\frac{y_{i,t}}{L_{i,t}}$$ (33)

$$\lambda_{t}^{e}q_{t}^{K}=\beta_{e}\lambda_{t+1}^{e}[\alpha_{1}mc_{i,t+1}\frac{y_{i,t+1}}{K_{i,t}}+q_{t+1}^{K}(1-\delta_{K})]+\lambda_{t}^{b}m_{b}\frac{q_{t+1}^{K}\pi_{t+1}}{R_{t}^{d}}$$ (34)

$$p_{t}^{E}+\phi_{1}{\left(e_{i,t}\right)}^{\phi_{2}}+tax_{t}^{CO_{2}}(1-e_{i,t})\varphi +p_{t}^{sub}\frac{e_{i,t}}{\varphi {\left(EN_{i,t}\right)}^{2}}=(1-\alpha_{1}-\alpha_{2})mc_{i,t}\frac{y_{i,t}}{EN_{i,t}}$$ (35)

$$\phi_{1}\phi_{2}{\left(e_{i,t}\right)}^{\phi_{2}-1}EN_{i,t}-tax_{t}^{CO_{2}}\varphi EN_{i,t}=p_{t}^{sub}{\left(\varphi EN_{i,t}\right)}^{-1}$$ (36)

### 3.3. Government departments

Revenue to the government includes labor taxes, investment taxes, energy taxes, carbon emission taxes, and government debt. Government expenditure include transfers to residents, subsidies for carbon emissions reduction to the enterprise sector, special expenditure on environmental protection, and the repayment of interest on debt from the previous period. Thus, the government’s budget constraints at time t can be expressed as follows:

$$\tau^{L}w_{t}L_{t}+\tau^{K}q_{t}^{K}I_{t}^{N}+\tau^{E}p_{t}^{E}EN_{t}+tax_{t}^{CO_{2}}+b_{t}^{g}=G_{t}$$ (37)

$$G_{t}=b_{t-1}^{g}\frac{R_{t-1}^{d}}{\pi_{t}}+G_{t}^{T}+G_{t}^{sub}+G_{t}^{E}$$ (38)

### 3.4. Market clearing

When the economic system reaches equilibrium, each economic actor achieves optimization, and the product and lending markets are cleared. In the product market, the aggregate demand for the entire market comprises the consumption and investment of representative households, consumption and carbon abatement costs of intermediate manufacturers, government transfers to residents, and government special expenditure on environmental protection. The aggregate supply is the output of final goods. Thus, economic resource constraints can be expressed as

$$Y_{t}=C_{t}^{h}+C_{t}^{e}+I_{t}+ERC_{t}+G_{t}^{E}$$ (39)

In the credit market, the supply side comprises representative households, whereas the demand side comprises entrepreneurs and governments. Thus, the equilibrium conditions for deposits and loans are:

$$D_{t}=b_{t}^{e}+b_{t}^{g}$$ (40)

$$\ln p_{t}^{sub}=(1-\rho_{p^{sub}})\ln\bar{p^{sub}}+\rho_{p^{sub}}\ln p_{t-1}^{sub}+\varepsilon_{p^{sub},t},\varepsilon_{p^{sub},t}∼N(0,\;\sigma_{p^{sub}}^{2})$$ (41)

$$\ln G_{t}^{E}=(1-\rho_{G^{E}})\ln\bar{G^{E}}+\rho_{G^{E}}\ln G_{t-1}^{E}+\varepsilon_{G^{E},t},\varepsilon_{G^{E},t}∼N(0,\;\sigma_{G^{E}}^{2})$$ (42)

where $\varepsilon_{p^{sub},t}$ and $\varepsilon_{G^{E},t}$ are stochastic disturbances, $\sigma_{p^{sub}}^{2}$ and $\sigma_{G^{E}}^{2}$ is the standard deviation of the two.

## 4. Parameter calibration

The DSGE model has been the dominant macroeconomic research method over the past 40 years. The first step in the application of the DSGE model is the construction of a theoretical model, followed by structural parameter estimation and dynamic simulation. There are three main methods of structural parameter estimation: calibration, maximum likelihood estimation (MLE), and Bayesian estimation (BE). The calibration method mainly refers to previous research results to determine the parameters of the model. The maximum likelihood estimation method and Bayesian estimation method estimate the parameters of the model using actual statistical or observational data. As the main focus of this study was theoretical research and mechanism analysis, we referred to Kydland and Prescott [48] and used calibration methods to determine the model parameters.

### 4.1. Parameters of households

We set the subjective discount rate *β*_*h*_ of representative households as 0.99, referring to the setting of Wang et al. [46]. We normalized the response parameters of the negative utility of labor *μ*_*L*_ and the negative utility of carbon emissions *μ*_*E*_ to 1 because they do not affect the model dynamics. Blanchard and Galí [49] set the inverse of labor supply elasticity *η* to 1.03 and 1.22; we set it to 1.2. The energy supply elasticity *ν* was set to 1.5, according to Xiao et al. [42] and Pan [50]. We set the coefficient of the adjusted investment cost *ψ*_*I*_ to 1.8, according to Xiao et al. [42].

### 4.2. Parameters of enterprises

Regarding the subjective discount rate of entrepreneurs, Iacoviello [51] argues that the discount factor of lenders is lower than that of depositors; that is, *β*_*e*_<*β*_*h*_. Thus, this study took it as 0.98, according to the settings of Wang et al. [46]. Regarding the technical parameters of production, the capital output elasticity *α*_1_ was taken as 0.33, according to Nalban [52], while the labor output elasticity *α*_2_ was taken as 0.58, according to Pop [53]. Regarding the parameters of the labor efficiency functions *d*_0_, *d*_1_, and *d*_2_, we referred to Heutel [43], Annicchiarico and Di Dio [44], with values of 1.395 × 10^ (−3), −6.6722 × 10^ (−3), and 1.4647 × 10^ (−8), respectively. The two coefficients of emission reduction costs of *ϕ*_1_ and *ϕ*_2_ were taken as 0.185 and 2.8, respectively, according to Annicchiarico and Di Dio [44]. The carbon emission factor per unit of energy was set to 0.6 by according to Xiao et al. [42]. The capital depreciation *δ*_*K*_, substitution elasticity of intermediate goods *θ*, and price stickiness coefficient *ξ* were taken as 0.025, 6, and 0.75, respectively, according to conventional values. The financing constraint coefficient *m*_*b*_ was set to its base value of 0.6, and compared with cases of weak financing constraints (*m*_*b*_ = 0.9) and strong financing constraints (*m*_*b*_ = 0.3).

### 4.3. Relevant environmental parameters

In this study, the natural attenuation coefficient of the carbon pollutant *δ*_*SC*_ was set to 0.005 by referring to Nordhaus [54] and Falk and Mendelsohn [55]. As for the effectiveness coefficient of government special expenditure on environmental protection *χ*, Angelopoulos et al. [56] set it at 0.6, 1.5, and 5; in this study, it was set at 1.16.

### 4.4. Parameters of government policies

Huang and Zhu [57] set the labor tax rate *τ*^*L*^ and investment tax rate *τ*^*K*^ to 0.051 and 0.266, respectively, which are consistent with the present study. According to the Notice of the Ministry of Finance and the State Administration of Taxation on the Relevant Policies on the Streamlining and Combination of Value-added Tax Rates (2017) of China, the rate of taxpayers selling liquefied petroleum gas, natural gas, gas, and coal products for residential use is 11% (https://www.chinatax.gov.cn/n810341/n810755/c2590976/content.html). Thus, the energy tax rate *τ*^*E*^ was set at 11% in this study. According to the Final Account of National General Public Budget Expenditure in 2020 published by the Ministry of Finance of China, the proportion of energy conservation and environmental protection expenditure to total fiscal expenditure after deducting pollution reduction expenditure was 2.88%; thus, this study set the proportion of government special expenditure on environmental protection to total fiscal expenditure at 2.88%. Meanwhile, from data on the share of transfer expenditure to residents—such as social insurance welfare benefits, pensions, old-age pensions, unemployment benefits, relief payments, and various subsidies—to total fiscal expenditure is approximately 10.5% (http://yss.mof.gov.cn/2020zyjs/); therefore, this study sets the share of transfer payments to total fiscal expenditure at 10.5%. Cai et al. [27] set the carbon emission reduction allowance rate *p*^*sub*^ to 5%, 10%, and 15%; in this study, it was set to 3%.

## 5. Impulse response analysis

### 5.1. The dynamic effects of environmental expenditure shocks

#### 5.1.1. Dynamic effects of emission reduction subsidy rates

**Fig 2:**
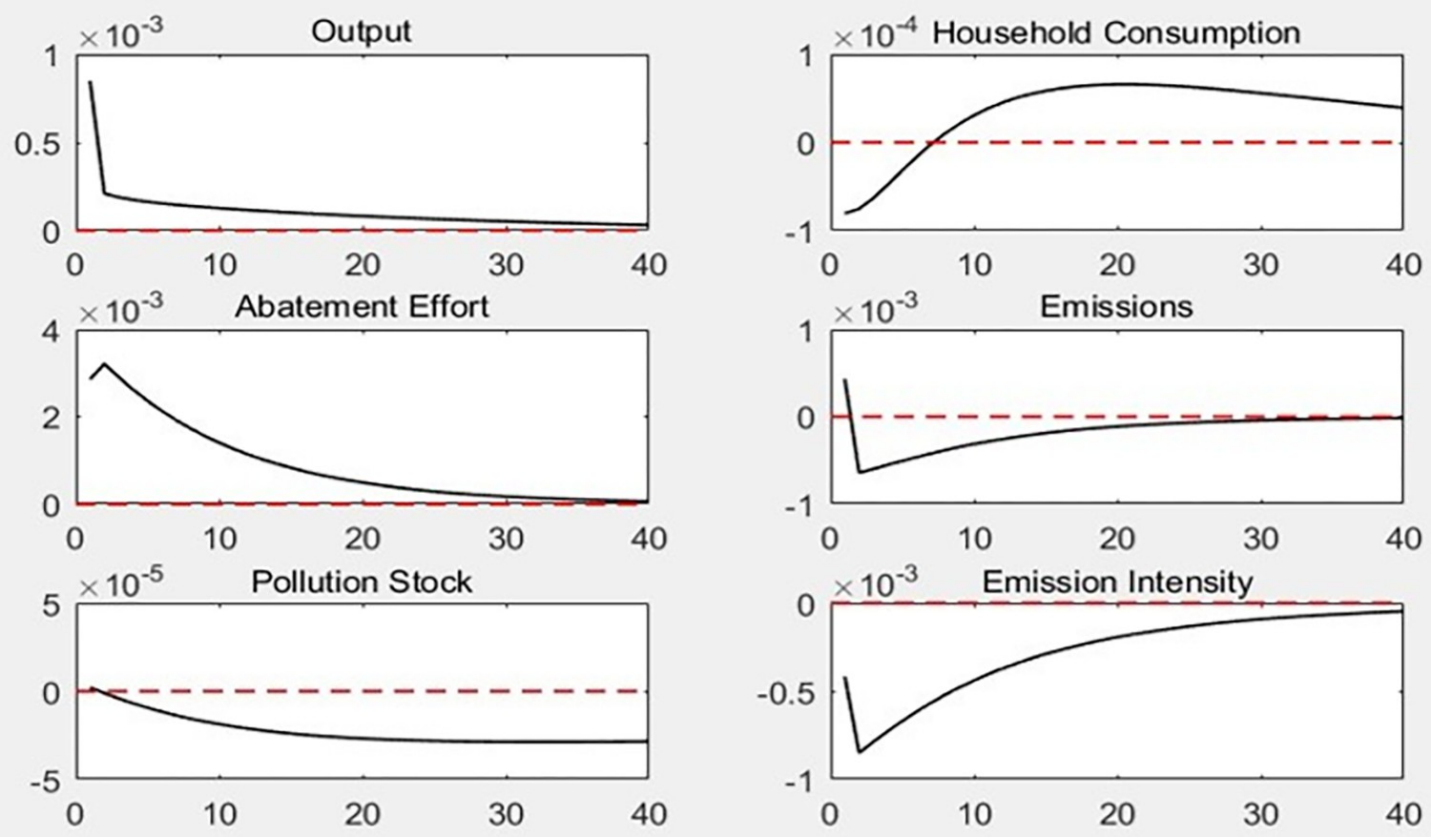
Dynamic effect of carbon emission reduction on subsidy rates.

Fig 2 illustrates the dynamic path of the impact of the 1% carbon emission reduction subsidy rate impact on each macro variable. Increasing carbon emission reduction subsidy rate not only reduces carbon emissions, pollution stock, and carbon emission intensity, but also increases the total economic output. In addition, the increase in the carbon emissions reduction subsidy rate was first negative and then positive for household consumption.

The dynamic effects of the carbon emission reduction subsidy rate impact are transmitted through three main mechanisms. (1) The increase in government subsidies for carbon emission reduction directly enables enterprises to obtain more liquidity, which promotes production and increases output to increase the total economic output. At the same time, subsidies increase the net profit of enterprises and improve their balance sheets, which enables them to obtain more loans and further increase output. (2) An increase in the emission reduction subsidy rate can both increase the net profit of enterprises and improve their financing ability, which will have an incentive effect on enterprises’ carbon emission abatement efforts, prompting them to improve their emission reduction ability by upgrading equipment and technology as well as improving management, which will have a positive effect on carbon emission reduction and result in decreased carbon emissions and pollution stock, thereby decreasing carbon emission intensity. (3) As carbon emissions reduction subsidies are part of government fiscal expenditure, increasing subsidies leads to a relative reduction in government transfer payments and special expenditure on environmental protection. Meanwhile, a decrease in government transfer payments directly reduces the income level of households, which in turn reduces the consumption of residents; however, as the level of total economic output increases, the increase in demand for labor by manufacturers raises the wage income of the household sector, which in turn raises household consumption.

#### 5.1.2. Dynamic effects of government special expenditure on environmental protection

**Fig 3:**
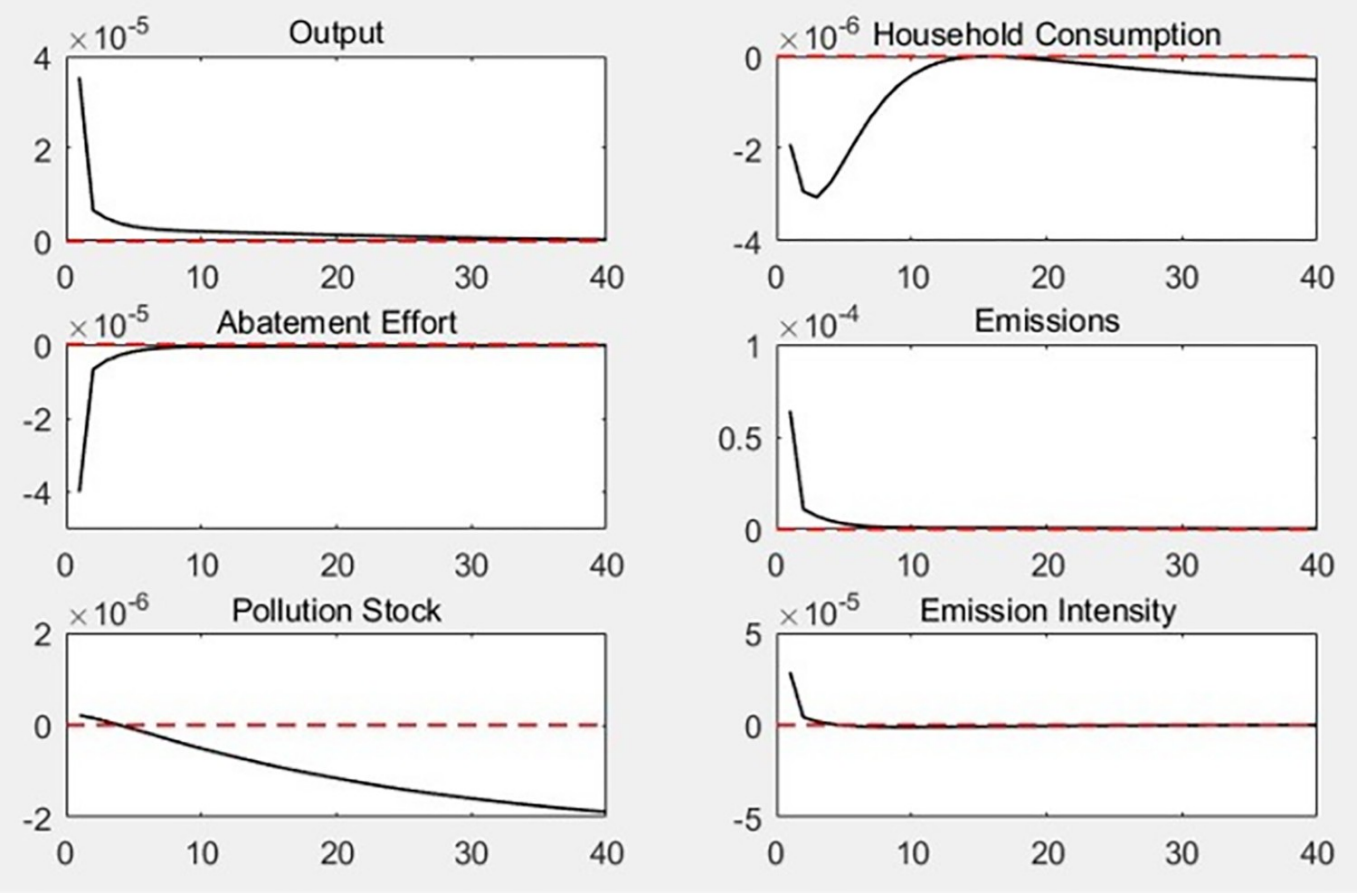
Dynamic effects of government special expenditure on environmental protection.

Fig 3 shows the dynamic change path of each macro variable under the impact of 1% government special expenditure on environmental protection. First, higher government special expenditure on environmental protection not only reduces the carbon emissions and carbon emission intensity of enterprises but also reduce the total economic output. Furthermore, the impact on the pollution stock first increases and then decreases. Furthermore, its effect on pollution shock shows an increase, followed by a decrease. Second, increasing government spending on pollution prevention and control negatively affects household consumption.

The dynamic effects of the impact of government special expenditure on environmental protection are mainly mediated through the following transmission mechanisms. (1) An increase in government special expenditure on environmental protection directly reduces the pollution stock and improves labor efficiency, promoting an increase in total economic output. Simultaneously, special government expenditure on environmental protection reduce enterprises’ efforts to reduce carbon emissions, making them subject to lower environmental pressures and leading to an increase in scale and further output. (2) The increase in government spending on environmental protection reduces enterprises’ carbon emission abatement efforts, leading to an increase in new carbon emissions and carbon emission intensity; however, the overall trend of pollution stock decreases as government pollution control directly reduces such stock. (3) The impact of government spending on household consumption is similar to that of carbon emission reduction subsidies; as such, it is not specifically analyzed here.

### 5.2. Influence of financing constraints on policy effect

#### 5.2.1. Long term effects of financing constraints

In China, enterprises generally face financing constraints in their development processes. In particular, small and medium-sized enterprises find it difficult and costly to obtain financing. This has always been a crucial, yet difficult problem that governments have committed to solving. Therefore, including financing constraints in the model better reflects the actual situation of China’s economic development, with financing constraints playing an important role in the formation of long-term equilibrium. In this study, the degree of financing constraint *m*_*b*_ was taken as 0.9, 0.6, and 0.3, representing weak, medium, and strong financing constraints, respectively. As shown in Table 2, with the change in financing constraints *m*_*b*_, the steady-state values of the main economic and environmental variables also change significantly. For example, when enterprises are exposed to weak financing constraints (*m*_*b*_ = 0.9), the steady-state value of the total economic output is 2.5393, while when enterprises are exposed to strong financing constraints (*m*_*b*_ = 0.3), the steady-state value of total economic output ($\bar{Y}$) is 2.4415; that is, a decrease of 4%, with significant changes in the steady-state values of the other main variables. Moreover, the steady-state value of household consumption ($\bar{C^{h}}$) showed similar changes. However, the steady-state values of three environmental indicators, namely carbon emissions ($\bar{CO_{2}}$), pollution stock ($\bar{SC}$), and carbon emission intensity ($\bar{EI}$), changed in the opposite direction. Therefore, in the long run, financing constraints may have opposite effects on the total economic output and carbon emissions.

**Table 2:** Steady state values of variables under different financing constraints.

steady-state value of variables	Financing constraint degree		
	*m*_*b*_ = 0.9	*m*_*b*_ = 0.6	*m*_*b*_ = 0.3
$\bar{Y}$	2.5393	2.4890	2.4415
$\bar{C^{h}}$	2.0957	1.9788	1.8776
$\bar{e}$	0.3414	0.3384	0.3357
$\bar{CO_{2}}$	0.3525	0.3575	0.3619
$\bar{SC}$	70.3544	71.3622	72.2564
$\bar{EI}$	0.1388	0.14356	0.1482

#### 5.2.2. Shock effect of carbon emission reduction subsidy rate under different levels of financing constraints

**Fig 4:**
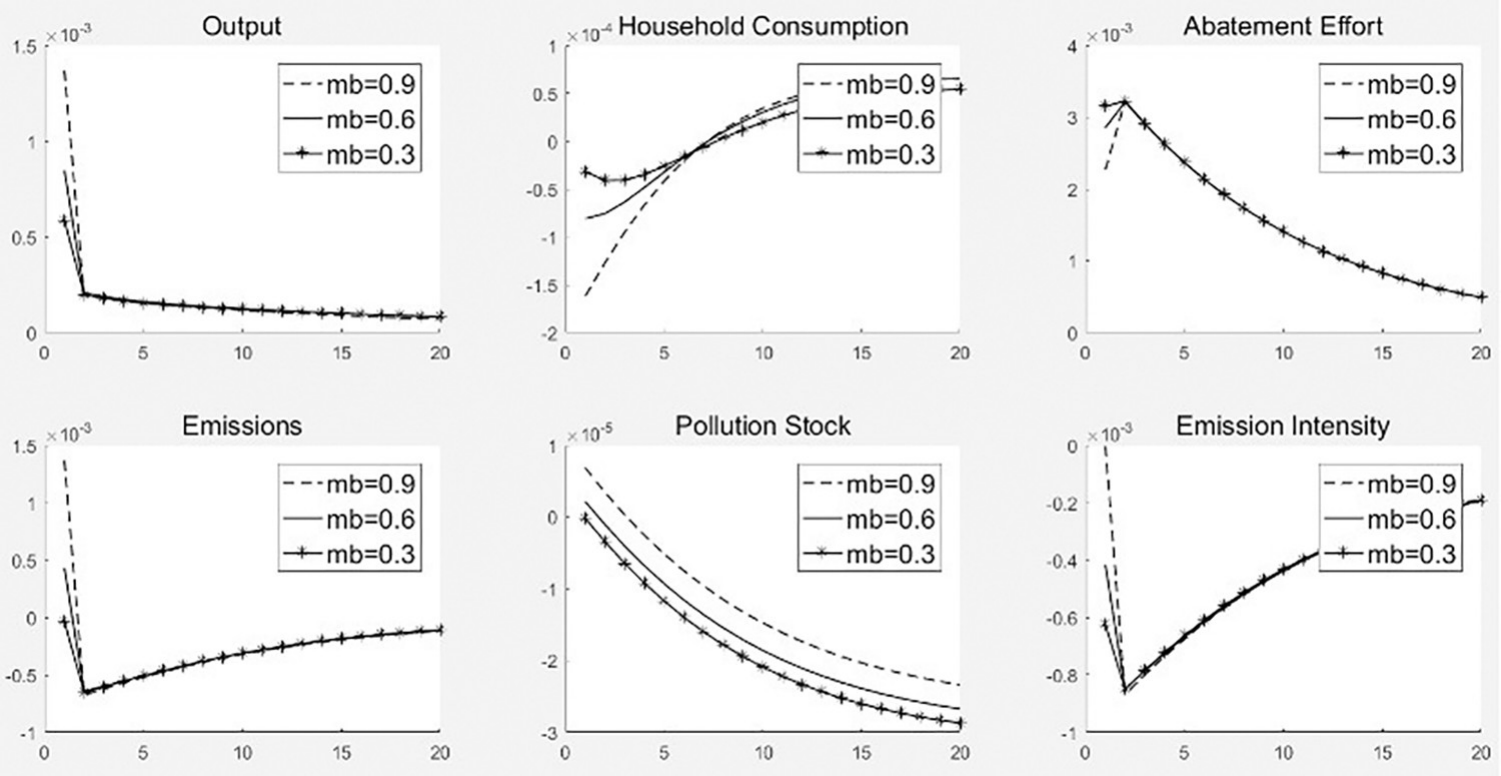
Shock effect of carbon emission reduction subsidy rate under different levels of financing constraints.

Fig 4 illustrates the dynamic path of the impact of the 1% carbon emission reduction subsidy rate on each macro-variable under the three financing constraints. Three main conclusions can be drawn from Fig 4. (1) The pull of carbon emission reduction subsidies on total economic output decreases as the degree of financing constraints increases. For example, under a 1% carbon emissions reduction subsidy rate shock, the total economic output in the first period increased under weak, medium, and strong financing constraints. However, the output effect was in the order of weak financing constraints > moderate financing constraints > strong financing constraints. (2) As the degree of financing constraint increases, the crowding-out effect of carbon emission reduction subsidies on household consumption decreases in the first period and becomes weaker in the second period as household consumption increases. For example, under a 1% carbon emission reduction subsidy rate shock in the first period, household consumption decreased under weak, medium, and strong financing constraints. The crowding out effect included weak, intermediate, and strong financing constraints. In the tenth period, household consumption increased under weak, medium, and strong financing constraints, and the crowding-out effect was weak financing constraints > medium financing constraints > strong financing constraints. (3) An increase in the degree of financing constraints causes a significant decrease in carbon emissions, pollution stock, and carbon emissions intensity under the same emissions reduction subsidy rate shock, resulting in a significant decrease in the emissions reduction effect.

Financing constraints affect the economic and environmental effects of emission reduction subsidy policies through the following mechanisms. (1) For total economic output, an increase in the carbon emission reduction subsidy rate increases the carbon emission reduction subsidies obtained by enterprises, increases the net profit of enterprises, and improves financing capacity. This makes the loans obtained by enterprises under weak financing constraints higher than those under medium financing constraints, resulting in an enhanced effect on the total economic output. In contrast, under strong financing constraints, enterprises obtain fewer loans than under medium financing constraints; thus, the promotional effect on total economic output is reduced. (2) As to household consumption, the increase in government spending on carbon emission reduction subsidies reduces the transfer payments received by households in the first period and decreases household consumption. However, an increase in the degree of financing constraints reduces the funds that households lend to enterprises, which in turn increases the funds available for household consumption. In the later period, the increase in the level of total economic output leads to an increase in households’ wage income, thereby causing an increase in household consumption. However, an increase in the degree of financing constraints results in greater dampening of the output-enhancing effect of carbon emission reduction subsidies and, thus, household consumption. (3) Financing constraints affect the emission reduction effect of emission reduction subsidy policies through the following mechanisms. On the one hand, increasing emission reduction subsidies can promote production enterprises to increase their emission reduction efforts, resulting in a decrease in pollution emissions and intensity; On the other hand, the increase in financing constraints leads to a decrease in loans obtained by production enterprises, which can only be addressed by reducing production scale, thereby further reducing pollution emissions and emission intensity.

#### 5.2.3. Shock effects of government special expenditure on environmental protection under different degrees of financing constraints

**Fig 5:**
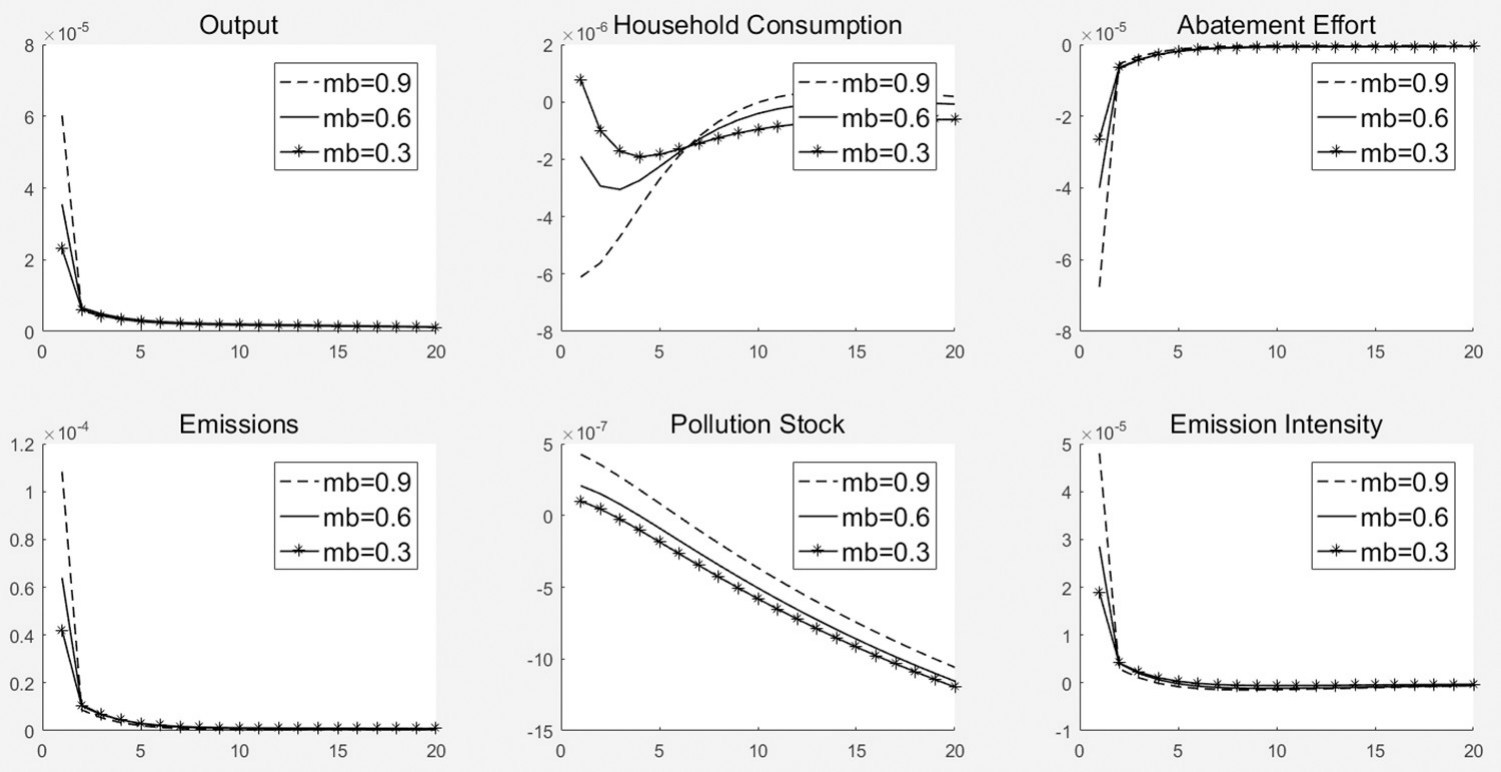
Shock effects of government special expenditure on environmental protection under different degrees of financing constraints.

Fig 5 illustrates the dynamic paths of the 1% impact of government special expenditure on environmental protection for each macro-variable under the three financing constraints. From Fig 5, we can draw three main conclusions. (1) As the degree of financing constraints increases, the pull of government special expenditure on environmental protection on total economic output decreases. For example, under a 1% impact of government special expenditure on environmental protection, the total economic output in the first period increased under weak, medium, and strong financing constraints. However, the output effect is in the order of weak financing constraints > medium financing constraints > strong financing constraints. (2) As the degree of financing constraints increases, the crowding-out effect of government expenditure on environmental protection shocks on household consumption decreases in the early stage and reduces the pull-on of household consumption in the later stage. For example, in the first period, household consumption decreases under both weak and medium financing constraints and increases under strong financing constraints, with a crowding-out effect of weak financing constraints > medium financing constraints > strong financing constraints. In the tenth period, household consumption rises under both weak and medium financing constraints and falls under strong financing constraints, with a crowding-out effect of weak financing constraints > medium financing constraints > strong financing constraints. (3) An increase in the degree of financing constraints significantly reduces carbon emissions, pollution stock, and carbon emissions intensity under the impact of equivalent government expenditure on environmental protection.

Financing constraints affect the economic and environmental effects of environmental protection special expenditure policies through the following mechanisms. (1) In terms of total economic output, increase the proportion of special expenditures on environmental protection means increasing government consumption, which is conducive to the growth of total output. However, financing constraints lead to a decrease in the credit funds available to production enterprises, which can only reduce production to cope, resulting in a decline in economic growth. Therefore, financing constraints suppress the output effect of environmental special expenditures. (2) As to household consumption, increase the proportion of expenditure on environmental protection can reduce the transfers received by households in the first period and decrease household consumption. However, an increase in the degree of financing constraints allows households to lend less money to firms, which in turn increases the funds available for household consumption. In the later period, the increase in the level of total economic output increases the wage income of households, which increases household consumption. Therefore, financing constraints have a promoting effect on household consumption in the early stage and a restraining effect in the later stage. (3) Financing constraints affect the emission reduction effect of environmental special expenditure policies through the following mechanisms. On the one hand, increasing the proportion of environmental protection expenditure can directly reduce the pollution stock, but it cannot incentivize enterprises to reduce emissions, leading to an increase in pollution emissions. On the other hand, financing constraints lead to a decrease in the credit funds obtained by production enterprises, resulting in a reduction in output and a decrease in emission intensity, pollution emissions, and environmental pollution stock. Therefore, financing constraints have promoted the pollution control effect of environmental protection expenditure policies.

### 5.3. Sensitivity analysis

**Fig 6:**
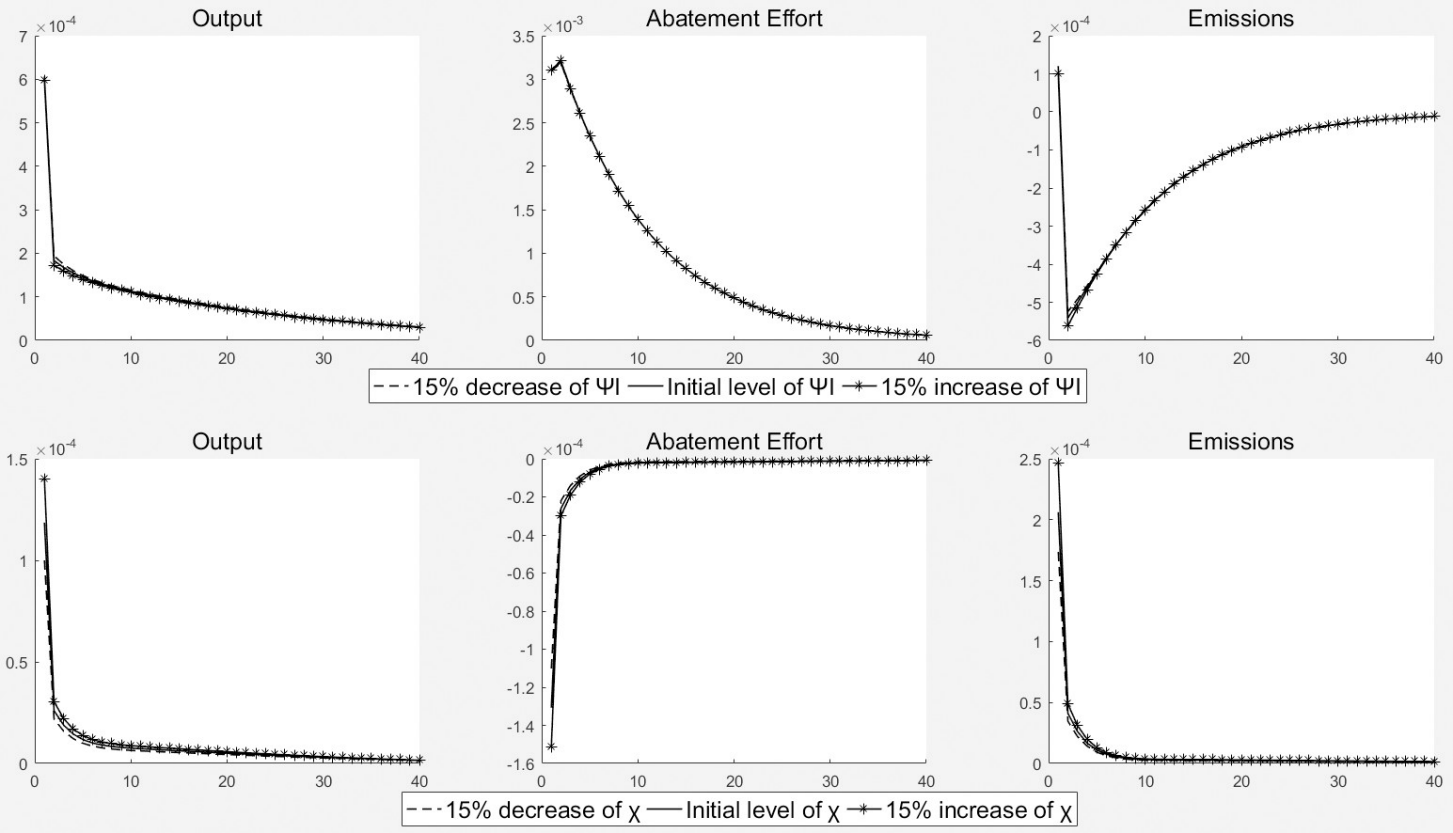
Results of sensitivity analysis.

In order to test the robustness of the model, sensitivity analysis was conducted in this paper. The specific approach is to adjust the important parameters of the model under the impact of two environmental expenditure policies, and determine its robustness by analyzing the changes in major economic and environmental variables. Fig 6 illustrates the impact of adjusting the coefficient of the adjusted investment cost *ψ*_*I*_ and effectiveness coefficient of government special expenditure on environmental protection *χ* by 15% on total output, corporate emission reduction efforts, and pollution emissions. It can be seen that the pulse response curve of the key variables only shows slight fluctuations, indicating that the model is very robust.

## 6. Conclusions

As a theoretical study of environmental policy, this study constructed an environmental DSEG model that incorporates two types of environmental expenditure (carbon emission reduction subsidies and government special expenditure on environmental protection) and uses the model to analyze the macroeconomic, economic, and environmental effects of carbon emission reduction subsidies and government expenditure on environmental protection. Furthermore, we compared the policy effects under different financing constraints. Based on this model, the following conclusions were drawn. (1) First, increasing carbon emission reduction subsidies in government expenditure not only increases total economic output but also motivates enterprises to increase emission reduction efforts and reduce pollution intensity and emissions, thereby reducing the inventory of environmental pollutants while balancing economic benefits and emission reduction. (2) Second, increasing the proportion of government special expenditure on environmental protection can not only promote output growth but also directly reduce the pollution stock in the environment. This has played a good role in post event "pollution control,” but will reduce the emission reduction efforts of enterprises, leading to an increase in their pollution emissions and intensity. (3) Third, the existence of financing constraints is not conducive to the growth of total output but increases the pollution control effect of emission reduction subsidies and pollution prevention expenditure.

The findings have several policy implications, including: (1) intensifying subsidies for green production enterprises and green projects to stimulate green production; (2) increasing government spending on pollution prevention and control, innovating the government’s approach to pollution control, and exploring pollution prevention and control spending models that can incentivize enterprises to reduce emissions and effectively cooperate with emission reduction subsidy policies; and (3) providing important tools for environmental governance that can provide financial support such as financing guarantees and credit subsidies for green enterprises and projects, which will promote the development of a green economy, and increasing the financing constraints of polluting enterprises and projects, which forces polluting enterprises to undergo green transformation. (4) Optimize the structure of fiscal expenditure and achieve greater results in promoting ecological environment governance. On the one hand, increasing the proportion of environmental expenditure in total fiscal expenditure promotes pollution reduction and environmental governance. On the other hand, emission reduction subsidy policies should be used in conjunction with special environmental protection expenditures. The emission reduction subsidy policy plays a role in preventing pollution in advance, while the special expenditure on environmental protection plays a role in controlling pollution afterwards.

The model constructed in this study also has shortcomings and cannot reflect the impact of environmental expenditure on different production sectors. In subsequent research, we will construct a heterogeneous production sector model that includes clean and polluting production sectors to study the impact of environmental expenditure on corporate behavior, pollution emissions, and economic output in different production sectors and further explore the impact of financing constraints on policy effectiveness. This will provide reference for policy departments to formulate credit policies targeting different industries.

## Supporting information

S1 File Data description. (DOCX)

S1 Appendix Competitive equilibrium. (DOCX)

S1 Data (XLSX)
