# Supplementary material for: The economic and environmental effects of China’s environmental expenditure under financing constraints
Source: PLoS One. 2024 Jul 12;19(7):e0305246. doi: 10.1371/journal.pone.0305246 (PMC11244846; doi:10.1371/journal.pone.0305246)
Supplement: S1 File — (DOCX) [file pone.0305246.s001.docx]

**Data Description**

regarding the explanation of the data. For the application of the DSGE model, the first step is the construction of the theoretical model, followed by parameter calibration, and finally, dynamic simulation. There are three main methods of structural parameter estimation: calibration, maximum likelihood estimation (MLE), and Bayesian estimation (BE). The calibration method mainly refers to previous research results to determine the parameters of the model. The maximum likelihood estimation method and Bayesian estimation method estimate the parameters of the model using actual statistical or observational data. As the main focus of this study was theoretical research and mechanism analysis, we mainly referred to Kydland and Prescott (1982) and used calibration methods to determine the model parameters. A few parameters were calculated using relevant data published on the official website of the Chinese Ministry of Finance. Therefore, there is no need to use other data. As shown in Table 1.

**Table 1.** Parameter calibration.

| **Par.** | **Parameters** | **Values** | **Sources** |
| --- | --- | --- | --- |
| $\beta$ | Household discount factor | 0.99 | [Wang et al. (2021)](#t31) |
| $\mu_{L}$ | Negative utility coefficient for labor | 1 | [Wu et al. (2011)](#t34) |
| $\mu_{E}$ | Negative utility coefficient for pollution emissions | 1 | [Wu et al. (2011)](#t34) |
| $\eta$ | Elasticity of household labor supply | 1.2 | [Blanchard & Galí (2010)](#t4) |
| $\nu$ | Energy supply elasticity | 1.5 | [Xiao et al. (2018)](#t26) |
| $\Psi_{I}$ | Quadratic portfolio adjustment cost coefficient | 1.8 | [Xiao et al. (2018)](#t26) |
| $\beta_{e}$ | Entrepreneur Discount factor | 0.98 | [Wang et al. (2021)](#t31) |
| $\alpha_{1}$ | Capital share of production function | 0.33 | [Nalban (2017)](#t24) |
| $\alpha_{2}$ | Labor share of production function | 0.58 | [Pop (2017)](#t29) |
| $d_{0}$ | Pollution damage function parameters | 1.395e-3 | [Heutel](#t5) (2012) |
| $d_{1}$ | Pollution damage function parameters | -6.6722e-6 | [Heutel](#t5) (2012) |
| $d_{2}$ | Pollution damage function parameters | 1.4647e-8 | [Heutel](#t5) (2012) |
| $\phi_{1}$ | Abatement cost function coefficient | 0.185 | [Annicchiarico & Di Dio (2015)](#t2) |
| $\phi_{2}$ | Abatement cost function parameter | 2.8 | [Annicchiarico & Di Dio (2015)](#t2) |
| $\delta_{K}$ | Capital depreciation rate | 0.025 | [Heutel](#t5) (2012) |
| $\theta$ | Substitution elasticity of intermediate goods | 6 | [Xiao et al. (2018)](#t26) |
| $\xi$ | Degree of price stickiness | 0.75 | [Heutel](#t5) (2012) |
| $\varphi$ | Emissions per unit of energy | 0.6 | [Xiao et al. (2018)](#t26) |
| $m_{b}$ | Degree of financing constraints | 0.3,0.6,0.9 | [Wang et al. (2021)](#t31) |
| $\delta_{PS}$ | Natural attenuation coefficient | 0.005 | [Nordhaus (1991)](#t25) |
| $\chi$ | Effectiveness coefficient of government spending on pollution treatment | 1.16 | [Angelopoulos et al. (2010)](#t1) |
| ${tax}^{L}$ | Labor tax rate | 5.1% | [Huang & Zhu (2015)](#t16) |
| ${tax}^{K}$ | Capital tax rate | 26.6% | [Huang & Zhu (2015)](#t16) |
| ${tax}^{E}$ | Steady-state value of energy tax | 11% | Real economic data |
| $\bar{{tax}^{M}}$ | Steady-state value of environmental tax | 0.06% | Real economic data |
| $\bar{s}$ | Steady-state value of emission reduction subsidy rate | 3% | [Cai et al. (2019)](#t6) |
| $\bar{{Ge}/G}$ | Steady-state ratio of pollution control expenditure | 2.88% | Real economic data |
