# Supplementary material for: The economic and environmental effects of China’s environmental expenditure under financing constraints
Source: PLoS One. 2024 Jul 12;19(7):e0305246. doi: 10.1371/journal.pone.0305246 (PMC11244846; doi:10.1371/journal.pone.0305246)
Supplement: S1 Appendix — (DOCX) [file pone.0305246.s002.docx]

Appendix: Competitive Equilibrium

$(C_{t}^{h})^{-1}=\lambda_{t}^{h}$ $\text{(A}1)$

$\mu_{L}L_{t}^{\eta}=\lambda_{t}^{h}(1-\tau^{L})w_{t}$ $\text{(A}2)$

$\text{(A}3)$

$1=\beta_{h}E_{t}\frac{\lambda_{t+1}^{h}}{\lambda_{t}^{h}}\frac{R_{t}^{d}}{\pi_{t+1}}$ $\text{(A}4)$

$\frac{1}{q_{t}^{K}(1-\tau^{K})}=1-\psi_{I}\left(\frac{I_{t}}{I_{t-1}}-1\right)\left(\frac{I_{t}}{I_{t-1}}\right)-\frac{\psi_{I}}{2}\left(\frac{I_{t}}{I_{t-1}}-1\right)^{2}+\beta_{h}E_{t}\frac{\lambda_{t+1}^{h}}{\lambda_{t}^{h}}\frac{q_{t+1}^{K}}{q_{t}^{K}}\psi_{I}\left(\frac{I_{t+1}}{I_{t}}-1\right)\left(\frac{I_{t+1}}{I_{t}}\right)^{2}$ $\text{(A}5)$

$I_{t}^{N}=I_{t}-\frac{\psi_{I}}{2}\left(\frac{I_{t}}{I_{t-1}}-1\right)^{2}I_{t}$ $\text{(A}6)$

$y_{t}\text{=}A_{t}K_{t-1}^{\alpha_{1}}(e_{t}^{L}L_{t})^{\alpha_{2}}EN_{t}^{1-\alpha_{1}-\alpha_{2}}$ $\text{(A}7)$

$e_{t}^{L}\text{=1}-\Gamma(CS_{t})$ $\text{(A}8)$

$\Gamma(CS_{t})=d_{0}+d_{1}CS_{t}+d_{2}(CS_{t})^{2}$ $\text{(A}9)$

$b_{t}^{e}=m_{b}\frac{q_{t+1}^{K}K_{t}\pi_{t+1}}{R_{t}^{d}}$ $\text{(A}10)$

$\text{(A}11)$

$K_{t}=(1-\delta_{K})K_{t-1}+I_{t}^{N}$ $\text{(A1}2)$

$(C_{t}^{e})^{-1}=\lambda_{t}^{e}$ $\text{(A}13)$

$\lambda_{t}^{e}=\beta_{e}\lambda_{t+1}^{e}\frac{R_{t}^{d}}{\pi_{t+1}}+\lambda_{t}^{b}$ $\text{(A}14)$

$w_{t}=\alpha_{2}mc_{t}\frac{y_{t}}{L_{t}}$ $\text{(A}15)$

$\lambda_{t}^{e}q_{t}^{k}=\beta_{e}\lambda_{t+1}^{e}\left[\alpha_{1}mc_{t+1}\frac{y_{t+1}}{K_{t}}+q_{t+1}^{k}(1-\delta_{K})\right]+\lambda_{t}^{b}m_{b}\frac{q_{t+1}^{K}\pi_{t+1}}{R_{t}^{d}}$ $\text{(A}16)$

$\text{(A}17)$

$\text{(A}18)$

$\pi_{t}^{*}=\frac{\theta}{\theta-1}\frac{M_{1t}}{M_{2t}}$ $\text{(A}19)$

$M_{1t}=\lambda_{t}^{h}mc_{t}Y_{t}+\beta_{e}\xi E_{t}(\pi_{t+i})^{\theta}M_{1,t+1}$ $\text{(A}20)$

$M_{2t}=\lambda_{t}^{h}Y_{t}+\beta_{e}\xi E_{t}(\pi_{t})^{\theta-1}M_{2,t+1}$ $\text{(A}21)$

$1=\xi\pi_{t}^{\theta-1}+(1-\xi)(\pi_{t}^{*})^{1-\theta}$ $\text{(A}22)$

$\Delta_{p,t}=\xi(\pi_{t})^{\theta}\Delta_{p,t-1}+(1-\xi)(\pi_{t}^{*})^{-\theta}$ $\text{(A}23)$

$y_{t}=\Delta_{p,t}Y_{t}$ $\text{(A}24)$

$CS_{t}=(1-\delta_{CS})CS_{t-1}+CO_{2t}-\chi G_{t}^{E}$ $\text{(A}25)$

$\text{(A}26)$

$EI_{t}=\frac{CO_{2t}}{y_{t}}$ $\text{(A}27)$

$\tau^{L}w_{t}L_{t}+\tau^{K}q_{t}^{k}X_{t}+\tau_{t}^{E}p_{t}^{E}EN_{t}\text{+}tax_{t}^{CO_{2}}\text{+}b_{t}^{g}=G_{t}$ $\text{(A}28)$

$G_{t}=b_{t-1}^{g}\frac{R_{t-1}^{d}}{\pi_{t}}+G_{1}^{T}+G_{1}^{sub}+G_{1}^{E}$ $\text{(A}29)$

$\text{(A}30)$

$\text{(A}31)$

$Y_{t}=C_{t}^{h}+I_{t}+C_{t}^{e}+ERC_{t}+G_{t}^{E}$ $\text{(A}32)$

$\text{(A}33)$

$D_{t}=b_{t}^{e}+b_{t}^{g}$ $\text{(A}34)$

$\log A_{t}=(1-\rho_{A})\log\overline{A}+\rho_{A}A_{t-1}+\varepsilon_{t}^{A}$ $\text{(A}35)$

$\ln tax_{t}^{CO_{2}}=(1-\rho_{tax^{CO_{2}}})\ln\overline{tax_{t}^{CO_{2}}}+\rho_{tax^{CO_{2}}}\ln tax_{t-1}^{CO_{2}}\text{+}\varepsilon_{tax^{CO_{2}},t}$ $\text{(A}36)$

$\ln\tau_{t}^{EN}=(1-\rho_{\tau^{EN}})\ln\overline{\tau^{EN}}+\rho_{\tau^{EN}}\ln\tau_{t-1}^{EN}\text{+}\varepsilon_{\tau^{EN},t}$ $\text{(A}37)$

$\ln G_{t}^{T}=(1-\rho_{G^{T}})\ln\overline{G^{T}}+\rho_{G^{T}}\ln G_{t-1}^{T}\text{+}\varepsilon_{G^{T},t}$ $\text{(A}38)$

$\ln p_{t}^{sub}=(1-\rho_{p^{sub}})\ln\overline{p^{sub}}+\rho_{p^{sub}}\ln p_{t-1}^{sub}\text{+}\varepsilon_{p^{sub},t}$ $\text{(A}39)$

$\ln G_{t}^{E}=(1-\rho_{G^{T}})\ln\overline{G^{T}}+\rho_{G^{T}}\ln G_{t-1}^{E}\text{+}\varepsilon_{G^{T},t}$ $\text{(A}40)$

$\ln R_{t}^{d}=(1-\rho_{R^{d}})\ln\overline{R^{d}}+\rho_{R^{d}}\ln R_{t-1}^{d}\text{+}\rho_{\pi}(\ln\pi_{t}-\ln\overline{\pi}\text{)+}\rho_{Y}(\ln Y_{t}-\ln\overline{Y}\text{)+}\varepsilon_{R^{d},t}$ $\text{(A}41)$
